# Contribution of Obstructive Sleep Apnoea to Cognitive Functioning of Males With Coronary Artery Disease: A Relationship With Endocrine and Inflammatory Biomarkers

**DOI:** 10.3389/fnins.2022.899597

**Published:** 2022-07-18

**Authors:** Nijole Kazukauskiene, Naomi A. Fineberg, Aurelija Podlipskyte, Adomas Bunevicius, Nicolás Francisco Narvaez Linares, Marilou Poitras, Hélène Plamondon, Aiste Pranckeviciene, Julija Gecaite-Stonciene, Narseta Mickuviene, Giedrius Varoneckas, Julius Burkauskas

**Affiliations:** ^1^Laboratory of Behavioral Medicine, Neuroscience Institute, Lithuanian University of Health Sciences, Palanga, Lithuania; ^2^National Obsessive Compulsive Disorders Specialist Service, Hertfordshire Partnership University, NHS Foundation Trust, Welwyn Garden City, United Kingdom; ^3^Behavioural Neuroscience Group, School of Psychology, University of Ottawa, Ottawa, ON, Canada

**Keywords:** obstructive sleep apnoea, cognitive function, NT-pro-BNP, N-terminal pro-brain natriuretic peptide, free triiodothyronine (fT3), free thyroxine (fT4), high-sensitivity c-reactive protein (hs-CRP)

## Abstract

**Introduction:**

Our exploratory study aimed to determine whether obstructive sleep apnoea (OSA) could affect cognitive functioning in males with coronary artery disease (CAD), and whether such impact could be associated with changes in thyroid hormones and inflammatory marker regulation on cognitive functioning.

**Method:**

We evaluated different endocrine and inflammatory biomarkers, including free triiodothyronine [fT3], free tetraiodothyronine [fT4], N-terminal pro-B-type natriuretic peptide [NT-pro-BNP], and high-sensitivity C-reactive protein [hs-CRP] serum levels in 328 males ($\bar{x}$ = 57 ± 10 years), undergoing cardiac rehabilitation after an acute coronary event. Participants underwent full-night polysomnography and were classified in mild/non-OSA (*n* = 253) and OSA (*n* = 75) according to an apnoea-hypopnoea index ≥ 15 event/h. Cognitive functioning testing included the Digit Span Test, Digit Symbol Test (DSST), and Trail Making Test. Analyses of variance assessed the impact of OSA on cognitive functioning and possible relationships of fT3/fT4, NT-pro-BNP and with hs-CRP on cognitive measures.

**Results:**

Significant group (OSA, mild/non-OSA) × NT-pro-BNP (<157.0 vs. ≥157.0, ng/L) interactions were found for the DSST raw score (*F*_(2,324)_ = 3.58, *p* = 0.014). Decomposition of interactions showed that the DSST scores of the OSA group with NT-pro-BNP ≥ 157.0 ng/L (*M* = 33.2; SD = 8.1) were significantly lower, *p* = 0.031, than those of the mild/non-OSA with NT-pro-BNP < 157.0 ng/L (*M* = 37.7; SD = 8.9).

**Conclusion:**

These findings indicate that males with OSA and clinically elevated NT-pro-BNP levels experienced inferior psychomotor performance compared to those without OSA and reduced NT-pro-BNP levels.

## Introduction

Obstructive sleep apnoea (OSA) represents an independent risk factor for adverse cardiac outcomes and impaired cognitive functioning, especially in men (Zhang et al., 2020). Several unfavorable medical conditions have been associated with OSA, for example coronary artery disease (CAD), heart failure, atrial fibrillation, hypertension, stroke (Goudis and Ketikoglou, 2017; Li et al., 2018; Salman et al., 2020; Roderjan et al., 2022), metabolic dysfunction (Bonsignore et al., 2013), and neurocognitive impairment (Seda and Han, 2020). Risk factors for OSA include older age, being male, a family history of OSA, obesity and upper airway anatomical abnormalities (Tishler et al., 2003; Lindberg et al., 2017; Kumar et al., 2021). Data from young and middle-aged individuals show a relationship between OSA and poor cognitive performance, notably in attention, memory, and executive function (Bubu et al., 2020). In older adults, OSA is not associated with a specific type of cognitive impairment at cross-section; however, OSA is linked with the possible development of mild cognitive impairment or Alzheimer’s disease with symptomatic individuals who have a higher likelihood of associated disturbed sleep and cognitive impairment (Bubu et al., 2020).

A meta-analysis on addressing the impact of sleep disruption on inflammation processes, including changes in inflammatory cytokines [i.e., high-sensitivity C-reactive protein (hs-CRP)] found a strong relationship between these factors (Irwin et al., 2016). Indeed, numerous studies have supported inflammation induced by OSA to lead to damage of vascular endothelial cells, which can alter the form and function of arteries (Yu et al., 2018; Orrù et al., 2020; Maniaci et al., 2021). In turn, endothelial dysfunction resulting from such condition has been associated with various comorbidities such as cardiovascular disease, metabolic dysfunction, and most critically neurocognitive impairment (Daulatzai, 2015; Song et al., 2015). Alongside the changes mentioned above, increased concentrations of N-terminal pro-B-type natriuretic peptide (NT-pro-BNP) and hs-CRP commonly observed in individuals with CAD have been associated with impaired cognitive functioning and unfavorable CAD prognosis (Brozaitiene et al., 2016; Burkauskas et al., 2017).

Furthermore, OSA also represents a risk factor for changes in thyroid hormone secretion, with associated reduced concentrations of serum free triiodothyronine (fT3) and free tetraiodothyronine (fT4) themselves increasing the risk of developing CAD (Burkauskas et al., 2018). Examining thyroid function in *N* = 156 subjects suspected to experience OSA, Takeuchi et al. (2015) found a significant association between the mean apnoea duration and thyroid-stimulating hormone (TSH) secretion.

The consequence of OSA on the endocrine system appears to be mediated mainly by intermittent hypoxaemia, sympathetic activation, elevated blood pressure and increased inflammation and oxidative stress (Lavrentaki et al., 2019). The thyroid hormone is a major physiological regulator of brain development and function (Bernal and Nunez, 1995). Notably, current studies indicate that thyroid hormones have a unique role in the development and functioning of brain regions regulating mood and cognition (Burkauskas et al., 2020; Przybylak et al., 2021).

Studies have indicated a possible association between OSA and hypothyroidism, but the significance of thyroid function screening in individuals with OSA has been controversial. Additionally, while the effect of an interaction between OSA and such hormonal changes on cognitive function have been proposed (Barletta et al., 2019), such contention remains to be thoroughly investigated in a clinical setting.

Therefore, our exploratory study aimed to determine whether OSA interacting with fT3/fT4, NT-pro-BNP and hs-CRP affects specific aspects of cognitive functioning in males with CAD.

## Materials and Methods

### Study Participants

From January 2014 to December 2019, we invited *N* = 400 males with CAD to participate in this study. All the participants were part of a rehabilitation program at the hospital Palangos Klinika within 7 days after treatment for acute coronary syndrome [ACS, such as myocardial infarction (MI) or angina pectoris (AP)]. Our inclusion criteria were: (a) confirmed CAD diagnosis and recent ACS as identified by a study cardiologist; (b) no history of arrhythmic disorder and/or implantation of a cardioverter defibrillator; (c) comprehension of Lithuanian language; (d) aged 18–80. Our exclusion criteria included: (a) unstable cardiovascular condition (*n* = 32); (b) severe comorbidities (e.g., kidney failure, musculoskeletal disease) (*n* = 25); and (c) unwillingness to participate in the study (*n* = 15). Thus, the final sample consisted of *N* = 328 males with CAD (mean age 57 ± 10 years, range 33–80). According to the existing guidelines, all participants were subjected to standard evaluation and treatment for the secondary prevention of CAD (Gibbons et al., 2002; Piepoli et al., 2010; Fletcher et al., 2013; O’Gara et al., 2013).

### Study Procedure

Within 2 days of admission to the rehabilitation program and after providing written consent, all study participants were evaluated for clinical factors [i.e., New York Heart Association (NYHA) functional class, history of ACS, and angina pectoris class], thyroid hormones and other biomarkers (fT3, fT4, NT-pro-BNP, and hs-CRP) as well as demographic information (i.e., age, education, and marital status).

Cognitive functioning testing included the Digit Span Test (DST; Wechsler, 1981), Digit Symbol Test (DSST; Wechsler, 1981), and Trail Making Test Part A (TMTA; Strauss et al., 2006), measuring auditory attention, psychomotor performance and perceptual speed, respectively. These tests were chosen based on the recommendation provided by the National Institute of Neurological Disorders and Stroke-Canadian Stroke Network guidelines for short cognitive testing (Hachinski et al., 2006). They all have been previously validated and used in Lithuania (Bunevicius et al., 1999; Burkauskas et al., 2008). This particular testing battery has also proven to identify cognitive vulnerabilities in individuals with CAD (Burkauskas et al., 2018). Raw scores of each test were converted to a unified T-scores adjusted for age and education level according to the norms provided for a specific testing battery.

Within 3 days, participants underwent full-night polysomnography and were classified as mild/non-OSA (*n* = 253) and OSA (*n* = 75) according to an apnoea-hypopnoea index (AHI) ≥ 15 event/h (3% of desaturation or arousal).

Informed consent was obtained. The study protocol conformed to the ethical guidelines of the 1975 Declaration of Helsinki. The Kaunas Regional Biomedical Research Ethics Committee approved the study protocol (Protocol No. BE-2-21; P1-38/2007; P2-38/2007).

### Measures

#### Digit Span Test

The digit span test (DST) was used to measure participants’ auditory working memory (Wechsler, 1981). The test is divided into two parts: (a) participants are required to repeat numbers using the same numerical order that was previously presented to them; and (b) participants have to repeat digits in reversed order. The results of the DST represent two possible cognitive outcomes with scores ranging from 0 to 14. Higher scores on the first part represent better auditory rehearsal, while higher scores in the second part represent better auditory working memory.

#### Digit Symbol Test

The digit symbol test (DSST; Wechsler, 1981) examines an individual’s psychomotor ability, speed visual-motor coordination and incidental learning. In the first part of this test, participants must quickly assign the correct symbol to a random digit based on the code provided on a separate table. Scores of the test range from 0 to 93. Higher scores on the first part represent better psychomotor performance. On the second part, the code table is no longer provided for participants, and they have to recall which number matches each symbol. The number of pairs correctly recalled is used as a measure of incidental learning.

#### Trail Making Test-Part A

The main cognitive function measured with the trail making test- part A (TMTA; Strauss et al., 2006) is perceptual speed. In the TMTA version, the participant has to connect randomly displayed numbers in numerical order. Time competition is essential for this task; the faster, the better.

#### Polysomnography, Definition of Sleep Apnoea

Overnight fully attended polysomnography monitoring was performed with the “Alice 4” polysomnography System (Respironics Inc., Marietta, GA, United States) in the sleep laboratory using standard recording techniques according to the American Academy of Sleep Medicine and precise protocol of polysomnography monitoring (Iber, 2007). Intake of sedative medication was not allowed 48 h before the investigation. Airflow was monitored using a thermistor placed at the nose and mouth, and arterial oxygen saturation (SaO2) was recorded continuously with a pulse oximeter. Apnoea was defined as the disappearance of airflow for over 10 s; hypopnoea was defined as *a* ≥ 50% decrease in airflow lasting for >10 s associated with arousal or *a* ≥ 3% decrease in SaO2 from the baseline level. All forms of sleep-disordered breathing were noted, and apnoeas were classified as obstructive, central or mixed where the combination was seen. An OSA is *a* ≥ 10 s pause in respiration associated with ongoing ventilatory effort. A central apnoea is *a* ≥ 10 s pause in ventilation with no associated respiratory effort (Iber, 2007; Somers et al., 2008). A central apnoea is particularly common among individuals with heart failure or a stroke (Somers et al., 2008). The most common type of sleep apnoea is OSA, and much of the pathophysiologic understanding of sleep apnoeas relies on studies of OSA (Somers et al., 2008). In our study, the influence of central and obstructive events was not separately analyzed.

The AHI is the most commonly used characteristic of sleep apnoea. Although sleep apnoea syndrome includes both, a polysomnography abnormality and symptoms, its severity is often defined by AHI alone. The AHI was calculated as the total number of apnoea and hypopnoea episodes per hour of sleep. According to the American Academy of Sleep Medicine criteria (Iber, 2007), sleep apnoea was defined with usual clinical AHI thresholds: no sleep disordered breating, <5 event/h; mild, ≥5 to <15 event/h; moderate, ≥15 to <30 event/h; and severe, ≥30 event/h. We used different cut-off levels of AHI to improve comparability with earlier studies on sleep apnoea for adapting a definition of mild-to-severe (AHI ≥ 5) or moderate-to-severe (AHI ≥ 15) sleep apnoea.

#### Laboratory Tests

All participants had a blood sample collected on the second day after admission to the rehabilitation clinic. Venous blood samples were drawn after a minimum of 12 h overnight fast to evaluate thyroid hormones, NT-pro-BNP and hs-CRP concentrations. Blood was centrifuged, and serum was frozen at −70°C. Serum concentrations of fT3 and fT4 were analyzed using radioimmunoassay kit RIA (R-EW-125, Belgium). Normal fT3 concentrations range from 2.0 to 4.0 pg/mL; fT4, from 7.0 to 17.0 pg/mL. The serum NT-pro-BNP concentrations were determined using a radio-immunoassay method (Roche Cobas analyser, Roche Diagnostics, Switzerland). The normal serum concentration of NT-pro-BNP is considered to be <157 ng/L (Brozaitiene et al., 2016). Serum hs-CRP concentration was assessed using the chemiluminescent immunoassay method (Beckman Coulter Unicel DXC 600) with normal values of ≤0.3 mg/dL.

### Statistical Analysis

We performed statistical analysis using the Statistical Package for Social Sciences, SPSS Statistics for Windows, Version 22.0.0.0 (IBM SPSS Statistics for Windows, Version 22.0. Armonk, NY: IBM Corp). Data is expressed as a mean ± standard deviation (SD) for continuous variables, as a number (percentage) for qualitative variables, and as medians (25th–75th percentiles) for variables with non-normal distribution. The variable distribution of similarity to normal was assessed visually and using the Kolmogorov–Smirnov test. The data characteristics were compared between groups with OSA and without OSA were using Fisher’s χ^2^ test, for the parametric two-tailed Student’s *t*-test test or nonparametric Mann–Whitney U test. Cognitive test scores were converted into demographically adjusted T-scores based on age and education (Joy et al., 2000; Tombaugh, 2004). T-scores are normalized to have an average of 50 and a standard deviation of 10.

Several analyses of variance (ANOVA) were performed on cognitive functioning for (OSA vs. mild/non-OSA) group interaction with fT3/fT4 (≥0.206 vs. <0.206), NT-pro-BNP (<157.0 vs. ≥157.0, ng/L) and with hs-CRP (<0.39 vs. ≥0.39, mg/dL)(Brozaitiene et al., 2016). We used Benjamini-Hochberg adjustment for multiple comparisons in the cognitive function domain, setting the critical value for a false discovery rate at 0.15.

## Results

Baseline information on sociodemographic characteristics of the study participants are presented in Table 1, separated according to OSA status. In brief, the mean participants’ age was 57 ± 10, and *n* = 215 (65.5%) had hypertension. In total, *n* = 254 (49.0%) participants experienced an acute MI, *n* = 257 (78.0%) had II NYHA functional class, and the remaining *n* = 34 (10.0%) were within the III NYHA functional class. No subjects were classified as having IV NYHA functional class. Participants (*N* = 328) were divided into an OSA group (*n* = 75) and a non-OSA group (*n* = 253). Participants with OSA were significantly older (*p* = 0.002; *d* = 0.393, had a higher body mass index (*p* = 0.001; *d* = 0.487), and a higher number had hypertension (*p* = 0.017, *V* = 0.133; see Table 1). The ratio fT3/fT4 was higher in individuals with CAD and OSA (*p* = 0.001; *d* = 0.372). Furthermore, individuals with CAD and OSA had lower scores in the psychomotor speed (DSST score; *p* = 0.011; *d* = 0.359) domain.

**TABLE 1:** Characteristics of the sample (*N* = 328) and descriptive statistics by presence of obstructive sleep apnoea (OSA).

Subjects characteristics	Total (*N* = 328)	With OSA (*n* = 75)	Without OSA (*n* = 253)	*p*	t/χ^2^	Cohen’s d or Cramer’s V
Age (years), mean (SD)	56.9 (9.8)	59.7 (8.1)	56.1 (10.1)	**0.002**	3.20	0.393
Body mass index, mean (SD)	29.6 (4.4)	31.3 (4.9)	29.1 (4.1)	**0.001**	3.54	0.487
Education, n (%)				0.352	2.09	0.071
Up to 8 years	36 (11.0)	7 (9.3)	29 (11.5)			
Secondary school or gymnasium graduate	151 (46.0)	40 (53.3)	111 (43.9)			
College/university degree	141 (43.0)	28 (37.3)	113 (44.7)			
Smoking, n (%)	53 (16.2)	13 (17.3)	40 (15.8)	0.753	0.100	0.019
Diagnosis, n(%)				0.199	1.65	0.075
Angina pectoris	74 (22.6)	21 (28.0)	53 (20.9)			
Acute myocardial infarction	254 (77.4)	54 (72.0)	200 (79.1)			
NYHA class, n (%)				0.168	3.57	0.102
I	37 (11.3)	5 (6.7)	32 (12.6)			
II	257 (78.4)	59 (78.7)	198 (78.3)			
III	34 (10.4)	11 (14.7)	23 (9.1)			
Hypertension, n (%)	263 (80.9)	67 (90.5)	196 (78.1)	**0.017**	5.74	0.133
Medication, n (%)						
Nitrates	100 (30.5)	22 (29.3)	78 (30.8)	0.805	0.061	0.012
Beta-blockers	288 (87.8)	70 (93.3)	218 (86.2)	0.096	2.78	0.092
N-terminal pro-B-Type natriuretic peptide concentration (ng/L), median (25 and 75 percentiles)	261.8 (93.2–576.6)	223.0 (71.3–505.7)	271.7 (102.5–633.1)	0.443		
High-Sensitivity C-Reactive Protein concentration (mg/dL), median (25 and 75 percentiles)	0.25 (0.11–0.55)	0.29 (0.14–0.55)	0.25 (0.10–0.56)	0.400		
Free Triiodothyronine concentration (pg/mL), mean (SD)	2.95 (0.39)	3.03 (0.46)	2.93 (0.37)	0.064	1.88	0.240
fT3/fT4, mean (SD)	0.25 (0.07)	0.28 (0.09)	0.25 (0.07)	**0.001**	3.37	0.372
**Digit Span Test**						
Forward T score, mean (SD)	51.4 (11.5)	50.2 (11.0)	51.8 (11.6)	0.304	–1.03	0.142
Backward T score, mean (SD)	48.8 (10.2)	47.9 (9.5)	49.1 (10.4)	0.362	−0.91	0.120
**Digit Symbol Test**						
Raw T score, mean (SD)	35.5 (8.7)	33.2 (7.8)	36.2 (8.9)	**0.011**	−2.55	0.359
Pairs recalled correctly T score, mean (SD)	33.3 (20.1)	31.2 (20.5)	34.0 (19.9)	0.302	−1.03	0.139
**Trail Making Test**						
Test A Time T score, mean (SD)	40.3 (19.1)	38.2 (21.0)	41.0 (18.6)	0.271	−1.10	0.141

Univariate ANOVA was used to compare groups’ differences of specific aspects of cognitive functioning. As shown in Table 2, after Benjamini-Hochberg correction significant group (OSA, mild/non-OSA) × NT-pro-BNP (<157.0 vs. ≥157.0 ng/L) interactions were found for the DSST raw score (*F*_(3,324)_ = 3.58, *p* = 0.014). Decomposition of interactions showed that DSST score of the OSA group with NT-pro-BNP ≥ 157.0 ng/L (*M* = 33.2; SD = 8.1) were significantly lower (*p* = 0.031), than those of the mild/non-OSA with NT-pro-BNP < 157.0 ng/L (*M* = 37.7; SD = 8.9).

**TABLE 2:** Means differences of the cognitive functioning in the four groups stratified according to the presence of obstructive sleep apnoea and the level of NT-pro-BNP (high vs. low).

	Mild/non-OSA × NT-pro-BNP < 157, *n* = 87	Mild/non-OSA × NT-pro-BNP ≥ 157, *n* = 166	OSA × NT-pro-BNP < 157, *n* = 30	OSA × NT-pro-BNP ≥ 157, *n* = 45	*p* *	*F*	*p*-value
				
	1	2	3	4			
**Digit span test**							
Forward T score, mean (SD)	52.3 (11.4)	51.5 (11.7)	48.8 (9.7)	51.1 (11.8)	0.570	0.672	
Backward T score, mean (SD)	49.1 (9.9)	49.2 (10.7)	45.2 (10.4)	49.7 (8.6)	0.224	1.47	
**Digit symbol test**							
Raw T score, mean (SD)	37.7 (8.9)	35.3 (8.8)	33.3 (8.8)	33.2 (8.1)	**0.014**	3.58	1:4 (0.031)
Pairs recalled correctly T score, mean (SD)	37.0 (20.1)	32.3 (19.7)	34.1 (17.3)	29.2 (22.5)	0.161	1.73	
**Trail making test**							
Test A Time T score, mean (SD)	45.2 (13.2)	38.7 (20.5)	38.3 (19.2)	38.1 (22.4)	0.048	2.66	

*Statistically significant (p < 0.015) differences in bold based on Benjamini-Hochberg correction within the groups.

*OSA, obstructive sleep apnoea; NT-pro-BNP, N-terminal pro-B-type natriuretic peptide.*

We found a tendency for group (OSA, mild/non-OSA) × hs-CRP (<0.39 vs. ≥0.39, mg/dL) interaction with the DSST raw score (*F*_(3,323)_ = 2.93, *p* = 0.034; see Table 3).

**TABLE 3:** Means differences of the cognitive functioning in the four groups stratified according to the presence of obstructive sleep apnoea and the level of hs-CRP (high vs. low).

	Mild/non-OSA × hs-CRP < 0.39, *n* = 169	Mild/non-OSA × hs-CRP ≥ 0.39, *n* = 83	OSA × hs-CRP < 0.39, *n* = 44	OSA × hs-CRP ≥ 0.39, *n* = 31	*p* *	*F*	*p*-value
				
	1	2	3	4			
**Digit span test**							
Forward T score, mean (SD)	51.2 (11.4)	52.9 (12.0)	50.8 (11.2)	49.3 (10.9)	0.456	0.871	
Backward T score, mean (SD)	49.5 (11.1)	48.4 (8.9)	47.6 (10.0)	48.3 (9.0)	0.667	0.522	
**Digit symbol test**							
Raw T score, mean (SD)	36.6 (9.2)	35.4 (8.1)	34.2 (7.9)	32.0 (7.7)		2.93	1:4 (0.041)
Pairs recalled correctly T score, mean (SD)	35.4 (19.1)	30.7 (21.1)	30.3 (17.6)	32.4 (24.3)	0.245	1.39	
**Trail making test**							
Test A Time T score, mean (SD)	41.2 (20.0)	40.8 (15.3)	39.6 (19.6)	36.2 (23.0)	0.606	0.614	

*There were no statistically significant (p < 0.015) differences based on Benjamini–Hochberg correction within the groups.

*OSA, obstructive sleep apnoea; hs-CRP, high-sensitivity C-reactive protein.*

As shown in Table 4, there were no statistically significant differences based on Benjamini-Hochberg correction within the groups.

**TABLE 4:** Means differences of the cognitive functioning in the four groups stratified according to the presence of obstructive sleep apnoea and the level of fT3/fT4 (high vs. low).

	Mild/non-OSA × fT3/fT4 < 0.206, *n* = 75	Mild/non-OSA × fT3/fT4 ≥ 0.206, *n* = 178	OSA × fT3/fT4 < 0.206, *n* = 18	OSA × fT3/fT4 ≥ 0.206, *n* = 55	*p* *	*F*
				
	1	2	3	4		
**Digit span test**						
Forward T score, mean (SD)	51.4 (11.0)	51.9 (11.9)	52.7 (13.4)	49.2 (10.0)	0.474	0.837
Backward T score, mean (SD)	49.1 (10.2)	49.2 (10.5)	52.3 (5.9)	45.6 (9.1)	0.044	3.76
**Digit symbol test**						
Raw T score, mean (SD)	35.7 (8.8)	36.3 (8.9)	33.3 (7.9)	33.2 (7.9)	0.084	2.24
Pairs recalled correctly T score, mean (SD)	35.0 (18.3)	33.5 (20.6)	25.1 (22.5)	33.5 (19.5)	0.277	1.29
**Trail making test**						
Test A Time T score, mean (SD)	39.4 (20.9)	41.6 (17.5)	42.1 (14.9)	36.7 (22.8)	0.383	1.02

*There were no statistically significant (p < 0.015) differences based on Benjamini–Hochberg correction within the groups.

*OSA, obstructive sleep apnoea; fT3/fT4, free triiodothyronine/free tetraiodothyronine ratio.*

## Discussion

To our knowledge, this exploratory study is the first to identify whether OSA and its interaction with fT3/fT4, NT-pro-BNP and hs-CRP affect specific aspects of cognitive functioning. Our results showed an interaction between OSA and NT-pro-BNP, associated with specific features of cognitive functioning problems in males with CAD. In contrast, the contribution of fT3/fT4 and hs-CRP to specific aspects of cognitive functioning in participants experiencing OSA was not readily apparent.

A recent review from our group supported associations between discrete hormones and biomarkers and impairments in cognitive function in individuals with established CAD (Burkauskas et al., 2018). The most widely studied predictor of long-term outcomes in individuals with CAD having experienced ACS is NT-pro-BNP (Brozaitiene et al., 2016). Other studies have shown higher NT-pro-BNP serum concentrations associated with frequent apnoea or hypoxemia in individuals with sleep apnoea (Strehmel et al., 2016). However, a study by Cifçi et al. (2010) with *n* = 33 consecutive individuals with OSA did not detect any significant difference between the severity of OSA and serum NT-pro-BNP levels (Cifçi et al., 2010). A review addressing biomarkers of cardiovascular stress supported significant associations between NT-pro-BNP and the presence and severity of OSA, although data remained varied and conflicting (Maeder et al., 2016). In our study, significantly elevated NT-pro-BNP levels in individuals with OSA could support NT-pro-BNP’s role as endogenous marker of heart failure strongly associated with changes in cognitive functioning. Consistent with this, our earlier study found NT-pro-BNP to be negatively related to perceptual speed, independently of clinical risk factors and depressive symptoms in *n* = 278 individuals with CAD (Burkauskas et al., 2017). In contrast, other studies have considered multiple specific cognitive domains to be affected by OSA (Patel and Chong, 2021). Together, these findings point to NT-pro-BNP as a valuable marker in further elucidating causal factors associated with elevated cardiovascular risk in individuals with OSA.

Numerous studies have also supported serum biomarkers related to inflammation, such as hs-CRP, to be activated in individuals with OSA (Stanek and Brożyna-Tkaczyk, 2021). Inflammation and OSA are strongly related and linked to vascular morbidity (Rocchi et al., 2022). Notably, the observational study by Huang et al. (2016) suggested that elevated cytokines such as hs-CRP, are related to impaired inattention and vigilance abilities in children with OSA. In contrast, we did not find a relationship between OSA and hs-CRP mediating specific cognitive functioning aspects.

Whereas reduced fT3 concentrations have been associated with worse perceptual speed and inferior efficacy in completing cognitive tasks in individuals with stable CAD and endured ACS (Burkauskas et al., 2017), our study did not indicate OSA-related changes in fT3/fT4 concentrations to affect the investigated aspects of cognitive functioning.

Taken together, our findings support effects of OSA on certain areas of cognitive functioning performance, independently of other sleep disturbances, which may help explain the higher cardiovascular risk associated with sleep-disordered breathing, particularly in males (Marin et al., 2005). Future studies assessing sex-specific effects of OSA on cognitive functioning may offer essential insight on this research topic.

The present study has some strengths and limitations. The strengths of our study include well-validated tests for evaluating culturally free cognitive functioning and an acceptable sample size. Another unique aspect of the current study is the number of biomarkers analyzed. A cross-sectional design is a major limitation of our study, which prevented us from evaluating a causal relationship of fT3, fT4, NT-pro-BNP, and hs-CRP concentrations with cognitive functioning. The inclusion of individuals undergoing cardiac rehabilitation limits the generalizability of our findings to individuals with acute CAD. Also, our findings should not be applied to individuals with CAD above the age of 80, individuals with past cerebrovascular accidents, or individuals with severe somatic illnesses, as these were the study exclusion criteria. We also have to acknowldege that dividing groups by clinical norms of various biomarkers (namely fT3, fT4, NT-pro-BNP, and hs-CRP) might have created uneven distribution of study participants in each group, resulting in some of the groups being underpowered to detect changes. Thus, these results should be treated as preliminary, and have to be replicated in a larger sample size of individuals with CAD.

Despite these limitations, this is the first description of cognitive impairments in patients with OSA and its interaction with biomarkers. More specifically, it would be interesting for future studies to measure specific aspects of cognitive functioning tests a few months after the ACS. Furthermore, by directly addressing the possible underlying causes for the pathological changes seen in patients with OSA, it would also be interesting to assess the effect of continuous positive airway pressure therapy on the variation of biomarkers as well as on the progression of the cognitive function in the medium-long term follow-up. Understanding of other neurocognitive effects of fT3, fT4 NT-pro-BNP and hs-CRP should be elucidated in future studies employing other focused and specific cognitive functioning assessment probes because such knowledge could facilitate the identification of novel therapeutic strategies.

## Conclusion

Our results indicate that males with OSA who have clinically elevated NT-pro-BNP levels experienced inferior psychomotor performance, as measured by the DSST, compared to those without OSA and with lower NT-pro-BNP levels. These findings reflect a probable interaction between OSA and NT-pro-BNP, increasing the risk of producing cognitive functioning problems. However, the interaction effect must be confirmed in future studies with larger sample sizes. Our results underscore the importance of referring individuals with CAD and OSA for a comprehensive neuropsychological assessment when a high NT-pro-BNP level is suspected, considering that CAD individuals are more at risk of developing cognitive impairment. Cognitive functioning was not impacted by interaction between OSA and fT3/fT4 as well as hs-CRP.

## Data Availability Statement

The raw data supporting the conclusions of this article will be made available by the authors, without undue reservation.

## Ethics Statement

This study involving human participants were reviewed and approved by the Kaunas Regional Biomedical Research Ethics Committee which approved the study protocol (Protocol Nos. BE-2-21; P1-38/2007; P2-38/2007). All participants provided their written informed consent to enter this study.

## Author Contributions

JB, NM, and GV conceived and designed the study. JG-S, JB, and NK were responsible for the data collection and evaluation. AuP performed the statistical analyses. NK prepared the manuscript. NAF, NFNL, AB, NL, MP, HP, and AiP provided the critical revision and read and approved the final manuscript. All authors contributed to the article and approved the submitted version.

## Conflict of Interest

JG-S works as a consultant at FACITtrans. JB works as a consultant at Cronos. NF reports personal fees from Sun, Otsuka, Abbott, Lundbeck, Taylor and Francis, Oxford University Press, grants and non-financial support from ECNP, Shire, personal fees and non-financial support from College of Mental Health Pharmacists, nonfinancial support from RANZCP, Sun, RCPsych, CINP, Int Society of Behavioral Addiction, WHO, International College of Obsessive Compulsive Spectrum Disorders, BAP, Janssen, Int Forum of Mood and Anxiety Disorders, Wiley, grants from MRC, Wellcome, other from MHRA, outside the submitted work; and non-financial support from the EU COST Action. The remaining authors declare that the research was conducted in the absence of any commercial or financial relationships that could be construed as a potential conflict of interest.

## Publisher’s Note

All claims expressed in this article are solely those of the authors and do not necessarily represent those of their affiliated organizations, or those of the publisher, the editors and the reviewers. Any product that may be evaluated in this article, or claim that may be made by its manufacturer, is not guaranteed or endorsed by the publisher.
